# The gut microbiome and antibiotic resistome of chronic diarrhea rhesus macaques (*Macaca mulatta*) and its similarity to the human gut microbiome

**DOI:** 10.1186/s40168-021-01218-3

**Published:** 2022-02-09

**Authors:** Shengzhi Yang, Yu Liu, Nan Yang, Yue Lan, Weiqi Lan, Jinyi Feng, Bisong Yue, Miao He, Liang Zhang, Anyun Zhang, Megan Price, Jing Li, Zhenxin Fan

**Affiliations:** 1 Key Laboratory of Bioresources and Eco-Environment (Ministry of Education), College of Life Sciences, Sichuan University, Chengdu, Sichuan China; 2 Institute of Blood Transfusion, Chinese Academy of Medical Sciences, Chengdu, Sichuan China; 3 Institute of Qinghai-Tibetan Plateau, Southwest Minzu University, Chengdu, China; 4 Sichuan Key Laboratory of Conservation Biology on Endangered Wildlife, College of Life Sciences, Sichuan University, Chengdu, Sichuan China; 5 Sichuan Key Laboratory of Conservation Biology for Endangered Wildlife, Chengdu Research Base of Giant Panda Breeding, Sichuan Academy of Giant Panda, Chengdu, Sichuan China

**Keywords:** Gut microbiome, Chronic diarrhea, *Macaca mulatta*, *Lactobacillus*, Antibiotic resistome

## Abstract

**Background:**

Chronic diarrhea is a common disease causing morbidity and mortality of captive rhesus macaques (RMs, *Macaca mulatta*). Chronic diarrhea in RMs is typically characterized by long-term diarrhea and a weak response to antibiotic treatment. Diarrhea is also a common disease in humans and can cause death. However, the etiology of about half of diarrheal cases of humans is still unclear. Therefore, we performed shotgun metagenomic sequencing to characterize the differences in the gut microbiome and resistome of chronic diarrhea RMs and asymptomatic individuals.

**Results:**

Our results showed *Lactobacillus* spp. (mainly *L. johnsonii*, *L. reuteri* and *L. amylovorus*) were significantly depleted in chronic diarrhea RM guts compared to asymptomatic individuals (5.2 vs 42.4%). Functional annotation of genes suggested these *Lactobacillus* spp. carried genes involved in the adhesion of intestinal epithelial cells and production of bacteriocin. Chronic diarrhea RM guts also had a significantly greater abundance of many other gut bacteria, including mucin-degrading bacteria and opportunistic pathogens. The metabolic pathways of chronic diarrhea RM gut microbiome were enriched in aerobactin biosynthesis, while the metabolic pathways of asymptomatic RM gut microbiome were enriched in the production of short-chain fatty acids (SCFAs). Chronic diarrhea RM guts had a significantly greater abundance of antibiotic resistance genes (ARGs), such as *ermF*, *aph(3’)-IIIa*, *ermB*, and *floR*. The strains isolated from feces and tissue fluid of chronic diarrhea RMs had higher resistance rates to the majority of tested antibiotics, but not cephamycin and carbapenem antibiotics. Gut microbial composition comparisons showed that several captive nonhuman primate (NHP) guts were more similar to the guts of humans with a non-westernized diet than humans with a westernized diet. Chronic diarrhea RM gut microbiome was strikingly similar to rural-living humans with diarrhea and humans with a non-westernized diet than asymptomatic RMs.

**Conclusions:**

Our results suggested chronic diarrhea significantly altered the composition and metabolic pathways of the RM gut microbiome. The frequent use of antibiotics caused antibiotic resistance in chronic diarrhea RM gut microbiome with serious consequences for individual treatment and survival. The findings of this study will help us to improve the effective prevention and treatment of diarrhea in RMs.

Video Abstract

**Supplementary Information:**

The online version contains supplementary material available at 10.1186/s40168-021-01218-3.

## Background

Chronic diarrhea is a common disease of captive rhesus macaques (RMs, *Macaca mulatta*) [1, 2]. Chronic diarrhea in RMs is often manifested by chronic inflammatory response of the colon, and long-term and recurrent diarrhea, yet there has been no specific pathogen identified and antibiotics treatment is frequently ineffective [3]. Chronic diarrhea in RMs can cause dehydration, malnutrition, growth retardation, weight loss, weak immune response, and even death, resulting in great losses of captive RM breeding potential [2, 4]. This diarrheal disease of humans is also one of the most widespread diseases across the world, which threatens human health and is a particular threat for children who experience higher morbidity and mortality [5–7]. Compared to developed countries, the incidence rate of diarrhea is higher in developing countries due to poor hygiene and sanitation [7, 8]. However, the etiology of about half of human diarrheal cases cannot be clearly defined [9, 10].

The etiology of RM chronic diarrhea likely results from complex and combined reactions from the gut microbiome, environment, and genetic factors [2, 11]. However, the gut microbiome may be the key factor of susceptibility to diarrheal disease [12]. The animal gut is colonized by a mass of microorganisms that form a large and complex microecosystem [13–15]. A stable gut microbiome is important in resisting pathogens, strengthening immunity, and assisting in digesting the food of the host [12, 16, 17]. For instance, many complex polysaccharides in the diet, such as cellulose and resistant starch, are partially or completely indigestible for many animals, but these undigested polysaccharides can be fermented by the anaerobic gut bacteria and converted into short-chain fatty acids (SCFAs) to inhibit pathogen growth and provide energy for the host [18–21]. Consequently, there are likely links between changes in the gut microbiome and pathogen growth. Previous studies have reported increases of pathogens, viruses, or parasites in the guts of some RMs with diarrhea, such as *Helicobacter macacae*, *Campylobacter jejuni*, *Shigella flexneri*, *Yersinia enterocolitica*, *Strongyloides fulleborni*, Adenovirus, Calicivirus, and Rotavirus [22–26], yet it is widely believed that there are no specific pathogens responsible for diarrhea in RMs [3]. Due to shared phylogeny and physiological similarities between many nonhuman primates (NHPs) and humans, NHPs usually serve as important models in human disease research and drug development [27–29]. Therefore, because of many similarities of diarrheal disease, diarrheal RMs may serve as potential models in researching human diarrheal disease and aid in identifying the etiology of human diarrhea [22, 30].

Antibiotic treatment can effectively relieve acute diarrhea in some RMs [1]. However, one of the key characteristics of chronic diarrhea in RMs is the ineffectiveness of antibiotic treatments [3]. Antibiotics can effectively relieve diarrhea when diarrhea first manifests in RMs, but diarrhea could reoccur in some individuals sometime later. Treatment of antibiotics becomes ineffective in RM diarrhea along with long-term use of antibiotics. Additionally, the frequent abuse of antibiotics has caused the emergence of antibiotic-resistant bacteria, and multi-resistant bacteria further complicate the treatment of acute or chronic diarrhea [31, 32]. The increasing prevalence of antibiotic resistance is a significant threat to global health [33]. Many studies have reported antibiotic resistance profiles in breeding livestock and poultry [34–36], but antibiotic resistance profiles in captive RMs are still unclear. We speculate that there is a significant antibiotic resistance in the gut microbiome of chronic diarrhea RMs to weaken the effect of antibiotic treatment. Therefore, antibiotic-resistant phenotype and genotype could help us to assess antibiotic resistance and identify effective antibiotics to treat diarrhea in RMs.

Consequently, in this study, we used fecal metagenomes to comprehensively characterize gut microbial compositions and metabolic pathways of chronic diarrhea RMs, to quantify the resistome of RM gut microbiome, and to compare the gut microbial compositions between NHPs and humans. Our results indicated that *Lactobacillus* was considerably reduced in the gut of chronic diarrhea RMs. There was enrichment of aerobactin biosynthesis by *Escherichia coli* and reduced production of SCFAs in the gut microbiome of chronic diarrhea RMs. We found significant antibiotic resistance in the gut of chronic diarrhea RMs, including more abundant antibiotic resistance genes (ARGs) and higher resistance rates to antibiotics. Our results also showed that the gut microbiome of NHPs was more similar to humans with a non-westernized diet. These results can enhance understanding of the RM gut microbiome and antibiotic resistome to improve the diagnosis and therapy of chronic diarrhea.

## Methods

### Sample collection

The captive RMs in this study were from a breeding base in Meishan City of Sichuan Province, China. Although some RMs initially appeared mildly diarrheal symptoms, the diarrheal symptoms of most RMs disappeared after the first treatment of antibiotics. However, some RMs were still repeatedly diarrheal after repeated treatment with antibiotics and then developed chronic diarrhea. These chronic diarrhea RMs were characterized by long-term and recurrent diarrhea and no response to antibiotic treatment.

In this study, we collected 11 fresh feces of chronic diarrhea RMs and 18 fresh feces of asymptomatic RMs (control). All asymptomatic RMs and chronic diarrhea RMs lived in a similar environment and received the same diet. The chronic diarrhea RMs had been frequently treated using antibiotics including levofloxacin, metronidazole, gentamycin, cephalosporin, and florfenicol. Neither the asymptomatic nor the chronic diarrhea RMs had received antibiotic treatment for 30 days prior to sample collection. These fresh feces were collected using sterile instruments and then stored in sterile sampling tubes with ice packs to transfer to the laboratory. All samples used for shotgun metagenomic sequencing were finally stored at – 80 °C until processing. No RMs were hurt during the collection of feces, and our research had no impact on the health and welfare of RMs. The studied RMs continued their captive existence after the completion of our sample collection. Information of all studied RMs is summarized in Table S1.

### Metagenome sequencing and quality control of raw data

The total DNA of RM feces was extracted using Tiangen DNA Stool Mini Kit (TIANGEN Biotech Co., Ltd. China) according to the manufacturer’s protocol. The extracted DNA was quantified by the NanoDrop. DNA samples with concentration > 10 ng/μl and A260/A280 > 1.6 were used for library preparation. The concentration of the final library was > 5 ng/μl in a volume of 50 μl. After we completed Illumina sequencing (Novogene Co., Ltd. China), adapters and low-quality reads in raw data were removed by Trimmomatic [37] and RM’s potential sequences were removed by Bowtie2 [38] based on the RM reference genome (assembly Mmul_10).

### Assembly, functional prediction, and quantification of genes

The assembly of the metagenome was performed using MEGAHIT [39] with the option “--min-contig-len 300.” The gene was predicted using Prodigal [40] with the option “-p meta.” The non-redundant gene set was constructed using CD-HIT [41] with the option “-c 0.95 -aS 0.90.” The non-redundant genes were further translated into amino acid sequences. These amino acid sequences were aligned using DIAMOND [42] to the Carbohydrate-Active enZYmes (CAZy) database [43]. The amino acid sequences were also aligned to the Kyoto Encyclopedia of Genes and Genomes (KEGG) database using KAAS [44] using the default parameter and the UniProt database [45] using DIAMOND to predict function. The quantification of these non-redundant genes in each metagenome was performed using Salmon [46] with the option “--meta.” The total abundance of each gene type consisted of the total abundance of all genes mapped to the same gene type. The abundances of gene families and microbial metabolic pathways were assessed using HUMAnN2 [47] based on the ChocoPhlAn database and UniRef90 EC filtered database [48] and were normalized by CPM (count per million).

### Identification of taxa in metagenomes

The taxonomic labels of metagenomic sequences were assigned using kraken2 [49] with the option “--use-mpa-style.” The abundances of taxa were normalized by relative abundance. Differences in abundances of taxa, functional genes, and metabolic pathways were identified using LEfSe [50]. PCoA significance testing was performed using adonis. Differential abundance was detected using Wilcoxon’s rank-sum test with *p* < 0.05. Adonis significance testing and Bray-Curtis distances were analyzed using the vegan package of R.

### Identification and quantification of ARGs in metagenomes

To quantify the abundance of ARGs in gut microbiome, ARGs were quantified using ShortBRED [51]. The shortbred_identify.py script of ShortBRED produced a FASTA file of markers, using ARG sets from the comprehensive antibiotic resistance database (CARD) [52] as proteins of interest and UniRef90 sequences as reference proteins. The shortbred_quantify.py script of ShortBRED quantified the abundance of ARGs in metagenomes. The network between abundances of ARGs and gut microbiome was drawn using Cytoscape [53].

### Isolation and resistant phenotype testing of bacteria

We isolated the bacteria from feces and tissue fluid, analyzing their resistant phenotype to confirm the antibiotic resistance of the gut microbiome. The suspension liquid of fresh feces of asymptomatic and chronic diarrhea RMs and suspension liquid of tissue fluid of a dead diarrheal RM were cultured on MacConkey agar medium at 37 °C for 24 h. The bacterial colonies were selected and further cultured on tryptic soy agar (TSA) medium at 37 °C for 24 h. These isolates were identified by the 16S rRNA gene. All isolates were tested for resistant phenotypes to 12 antibiotics using Kirby-Bauer disk diffusion. The 12 antibiotics were cefotaxime (CTX), gentamycin (CN), florfenicol (FFC), tetracycline (TE), kanamycin (K), imipenem (IPM), ciprofloxacin (CIP), cefazolin (KZ), ofloxacin (OFX), cefoxitin (FOX), streptomycin (S), and ampicillin (AMP). The antibiotic susceptibility of isolates was interpreted by Performance Standards for Antimicrobial Susceptibility Testing (CLSI) (M100-S30). The *Escherichia coli* ATCC 25922 served as a quality control strain.

### Collection of public data

We analyzed the fecal metagenomes of humans and five species of NHPs to compare the gut microbiome of NHPs and humans. We downloaded the fecal metagenomes of captive *Macaca fascicularis* (*n* = 20) [54], *Pan troglodytes* (*n* = 18), *Gorilla gorilla gorilla* (*n* = 15) [55] and 80 healthy humans (41 individuals with a non-westernized diet and 39 with a westernized diet [56–62]) from public databases. NHPs samples were supplemented by six fecal metagenomes of captive or semi-captive *Macaca thibetana* that had been sequenced in our previous study. We also downloaded 67 fecal metagenomes of humans with inflammatory bowel diseases (IBD), encompassing 46 samples with Crohn’s disease (CD, a type of IBD) and 21 samples with ulcerative colitis (UC, a type of IBD) [63]. We then downloaded 11 fecal metagenomes from humans living rurally with acute diarrhea and 37 fecal metagenomes from urban humans with acute diarrhea from public databases [64]. These metagenomes were filtered using Trimmomatic and Bowtie2 to remove low-quality reads and hosts’ sequences. The taxonomic labels of these metagenomic sequences were assigned using kraken2. The abundances of taxa were normalized by relative abundance.

## Results

### The gut microbial composition of chronic diarrhea RMs and asymptomatic RMs

We compared the gut microbiomes of asymptomatic RMs and chronic diarrhea RMs to characterize differences in gut microbial composition. There were seven dominant phyla whose relative abundances were more than 1% in at least one sample, namely Firmicutes (17.9–87.0%), Bacteroidetes (3.1–36.0%), Proteobacteria (2.8–76.2%), Actinobacteria (1.2–24.9%), Spirochaetes (0.1–12.8%), Verrucomicrobia (0.03–15.65%) and Tenericutes (0.08–1.91%) (Fig. S1a). The relative abundances of Firmicutes and Spirochaetes in the guts of asymptomatic RMs were significantly greater than in chronic diarrhea RMs (Wilcoxon’s rank-sum test, *p* < 0.01). The relative abundances of Proteobacteria and Actinobacteria in the guts of chronic diarrhea RMs were significantly greater than asymptomatic RMs (Wilcoxon’s rank-sum test, *p* < 0.05 and *p* < 0.001, respectively) (Fig. S1b and c).

**Fig. 1:**
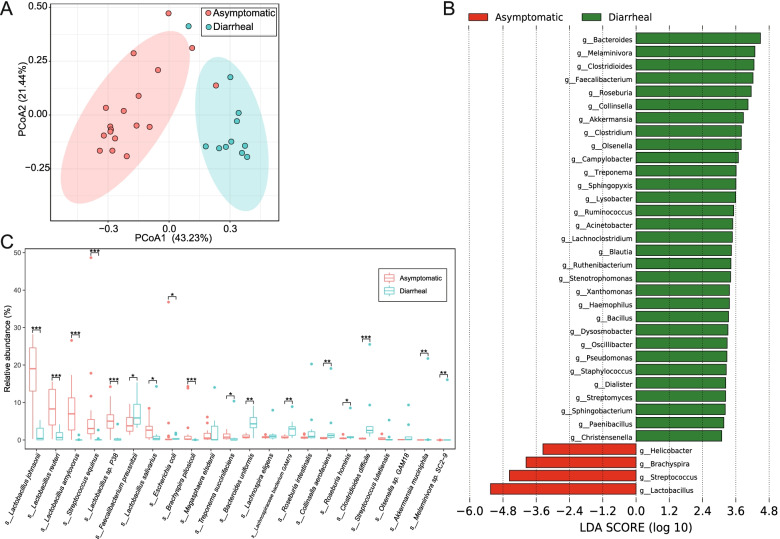
The gut microbiome composition of chronic diarrhea RMs and asymptomatic RMs. **a** PCoA plot based on Bray-Curtis distance of genus-level relative abundance profile (adonis, *R*^2^ = 0.3348, *p* = 0.001). **b** The difference of genus-level relative abundance by LEfSe (*p* < 0.05 and LDA > 3). **c** The comparison of main bacterial species. *p-value < 0.05; **p-value < 0.01; ***p-value < 0.001

A PCoA plot based on a genus-level relative abundance profile showed that axis 1 (PCoA1) explained 43.23% of the variability and axis 2 (PCoA2) explained 21.44% of the variability. The PCoA plot demonstrated that the samples of asymptomatic RMs and chronic diarrhea RMs were almost completely separated (Fig. 1a). We compared the gut microbiome of RMs with different ages and genders based on the PCoA plot, which exhibited all samples were separated by diarrheal disease rather than ages and genders (Fig. S1d and e). The guts of asymptomatic RMs were dominated by *Lactobacillus* (42.4% ± 19.9%), followed by *Streptococcus* (15.7% ± 16.2%), *Prevotella* (5.1% ± 3.6%), *Bacteroides* (4.2% ± 2.1%) and *Faecalibacterium* (3.7% ± 2.1%). The gut microbiome of chronic diarrhea RMs was enriched in *Bacteroides* (10.2% ± 5.3%), *Streptococcus* (8.5% ± 16.2%), *Faecalibacterium* (7.0% ± 4.2%), *Prevotella* (6.6% ± 5.0%) and *Lactobacillus* (5.2% ± 4.7%) (Fig. S1f). We compared the relative abundance of gut bacteria at genus level between asymptomatic RMs and chronic diarrhea RMs using LEfSe (Fig. 1b). Only four genera including *Lactobacillus*, *Streptococcus*, *Brachyspira*, and *Helicobacter* were significantly more abundant in the guts of asymptomatic RMs (*p* < 0.05 and LDA > 3). More genera, such as *Bacteroides*, *Clostridioides*, *Faecalibacterium*, *Roseburia*, *and Collinsella*, were more abundant in the guts of chronic diarrhea RMs (*p* < 0.05 and LDA > 3). In the guts of asymptomatic RMs, the dominant species included *Lactobacillus johnsonii* (17.2% ± 8.7%), *Lactobacillus reuteri* (8.6% ± 5.0%), *Lactobacillus amylovorus* (7.6% ± 6.7%), *Streptococcus equinus* (6.8% ± 11.0%), and *Lactobacillus* sp. P38 (5.3% ± 3.6%), which were significantly more abundant than in chronic diarrhea RM guts (*p* < 0.05) (Fig. 1c and Fig. S1g). *Faecalibacterium prausnitzii* (7.7% ± 4.2%), *Clostridioides difficile* (5.0% ± 6.8%), *Bacteroides uniformis* (4.6% ± 2.8%), *Roseburia intestinalis* (3.2% ± 5.6%), and *Collinsella aerofaciens* (3.0% ± 5.2%) were the most prevalent species in chronic diarrhea RM guts.

Due to mucin degradation likely leading to diarrhea, we also compared the relative abundance of mucin-degrading bacteria [65] in the guts of RMs. The relative abundances of these mucin-degrading bacteria including *Bacteroides thetaiotaomicron*, *Bacteroides vulgatus*, *Akkermansia muciniphila*, *Bifidobacterium bifidum*, *Bifidobacterium longum*, *Bifidobacterium breve*, and *Ruminococcus gnavus* in the guts of chronic diarrhea RMs were significantly greater than in asymptomatic RM guts (Fig. S1h).

### Functional comparison of the gut microbiome between chronic diarrhea RMs and asymptomatic RMs

Due to the greater abundance of *Lactobacillus* in asymptomatic RM guts, the potential contigs of *Lactobacillus johnsonii*, *Lactobacillus reuteri* and *Lactobacillus amylovorus* were screened out and annotated. *Lactobacillus johnsonii* harbored probiotic genes encoding lactate dehydrogenases, mucus-binding proteins (MUB), bile salt hydrolase (BSH), and bile salt transporter (BST). Similarly, these genes encoding lactate dehydrogenases, bacteriocin, bacteriocin ABC transporter, MUB, and BSH were identified in contigs of *Lactobacillus reuteri*. The genes encoding lactate dehydrogenases, MUB, and bacteriocin were also identified in contigs of *Lactobacillus amylovorus*.

**Fig. 2:**
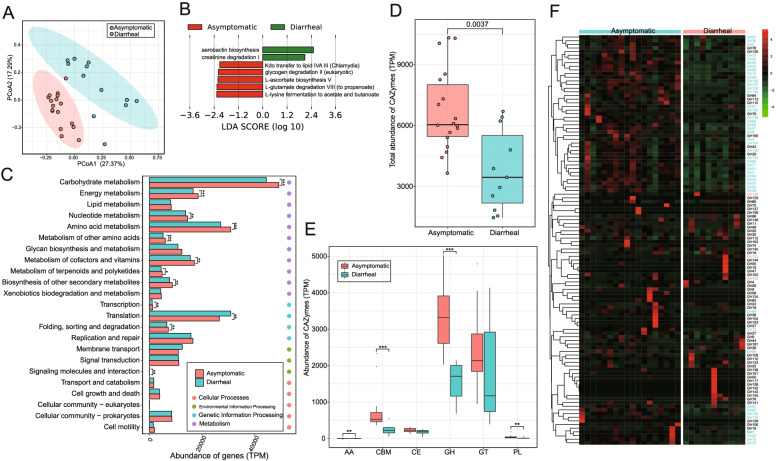
The functional comparison of gut microbiome between chronic diarrhea RMs and asymptomatic RMs. **a** PCoA plot based on Bray-Curtis distance of gene-family abundance (adonis, *R*^2^ = 0.19675, *p* = 0.001). **b** The difference of metabolic pathway abundance by LEfSe (*p* < 0.05 and LDA > 2). **c** The comparison of the main KEGG function. **d** The abundance comparison of total CAZymes. **e** The abundance comparison of GH (glycoside hydrolase), GT (glycosyl transferase), CBM (carbohydrate-binding module), CE (carbohydrate esterases), PL (polysaccharide lyases), and AA (auxiliary activities). **f** The distribution of the main GH family. The blue GHs means significantly more abundant in the gut microbiome of asymptomatic RMs and red GHs means significantly more abundant in the gut microbiome of chronic diarrhea RMs. *p-value < 0.05, **p-value < 0.01 and ***p-value < 0.001 in **c** and **e**

The abundances of microbial gene families and metabolic pathways were quantified using HUMAnN2 to compare the functional differences in the gut microbiome. A PCoA plot based on gene family abundance exhibited that the samples from asymptomatic RMs and chronic diarrhea RMs were almost completely spatially separated (Fig. 2a). Notably, the metabolic pathways of chronic diarrhea RM gut microbiome were enriched in aerobactin biosynthesis, largely due to *Escherichia coli*. The metabolic pathways of the asymptomatic RM gut microbiome were enriched in the production of SCFAs and L-ascorbate (Fig. 2b).

To further characterize the global functions of the gut microbiome, we predicted gene functions of the gut microbiome based on the KEGG database. The most abundant genes in the gut microbiomes of asymptomatic RMs and chronic diarrhea RMs were associated with metabolism, particularly carbohydrate metabolism, amino acid metabolism, metabolism of cofactors and vitamins, and energy metabolism (Fig. 2c). However, these genes involved in most metabolisms, such as carbohydrate metabolism, amino acid metabolism, metabolism of cofactors and vitamins, and energy metabolism, were significantly more abundant in the gut microbiome of asymptomatic RMs than in chronic diarrhea RM gut microbiome (*p* < 0.01). The majority of genes of carbohydrate metabolism, including butanoate metabolism, propanoate metabolism, pyruvate metabolism, and metabolism of starch and sucrose, were significantly more prevalent in the gut microbiome of asymptomatic RMs than chronic diarrhea RMs (*p* < 0.05) (Fig. S2a).

Due to the high abundance of genes associated with carbohydrate metabolism in the guts of RMs, we predicted carbohydrate-active enzymes (CAZymes) of the gut microbiome based on the CAZy database to further understand the activity of CAZymes of the gut microbiome. These CAZymes were identified from six classes of enzymes, namely glycoside hydrolase (GH), glycosyltransferase (GT), carbohydrate-binding module (CBM), carbohydrate esterase (CE), polysaccharide lyase (PL), and auxiliary activity (AA). Total genes of CAZymes in the asymptomatic RM gut microbiome were significantly more abundant than in chronic diarrhea RM gut microbiome (Fig. 2d). Notably, the genes encoding CBMs, GHs, and PLs were significantly more abundant in the gut microbiome of asymptomatic RMs (Fig. 2e). We found that GH13 was the most prevalent GH family in the guts of asymptomatic and chronic diarrhea RMs (Fig. 2f and Fig. S2b). However, a relative abundance of GH13 was significantly greater in the asymptomatic RM gut microbiome than in the chronic diarrheal RM gut microbiome. Additionally, most GH families, such as GH1, GH43, GH3, GH23, and GH25, were significantly more abundant in the asymptomatic RM gut microbiome.

### Comparison of gut microbiome between captive NHPs and humans

**Fig. 3:**
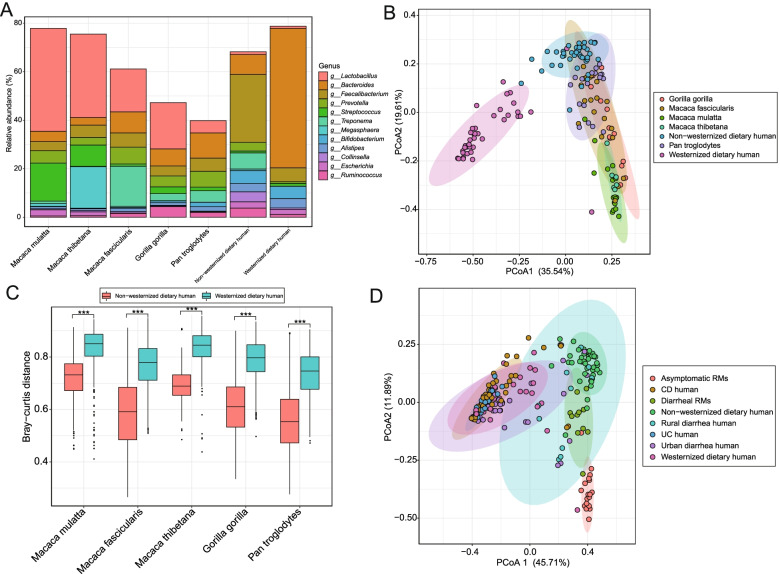
The comparison of gut microbiome between NHPs and humans. **a** The main bacterial genera in the gut of NHPs and humans. **b** PCoA plot based on Bray-Curtis distance of the genus-level relative abundance of gut microbiome between NHPs and humans (adonis, *R*^2^ = 0.55534, *p* = 0.001). **c** Bray-Curtis distance of the genus-level relative abundance of gut microbiome between NHPs and humans. ***p-value < 0.001 (**d**) PCoA plot based on Bray-Curtis distance of genus-level relative abundance profile between RMs and humans with acute diarrheal or IBD (adonis, *R*^2^ = 0.48215, *p* = 0.001)

We compared the gut microbial composition of asymptomatic RMs, captive *Macaca thibetana*, captive *M. fascicularis*, captive *Pan troglodytes*, captive *Gorilla gorilla*, humans with a non-westernized diet, and humans with a westernized diet. *Lactobacillus* was prevalent in the guts of all NHPs but was scarce in the guts of humans (Fig. 3a and Fig. S3a). The PCoA plot based on Bray-Curtis distance of genus-level relative abundance profiles exhibited that almost all samples of NHPs (particularly *M. fascicularis*, *P. troglodytes*, and *G. gorilla gorilla*) were spatially close to the samples of humans with a non-westernized diet (Fig. 3b). Differences in Bray-Curtis distances showed that the gut microbiome of captive *P. troglodytes* were more similar to both humans with a non-westernized diet and a westernized diet (Fig. S3b). Meanwhile, the gut microbiome of each captive NHP was more similar to humans with a non-westernized diet than humans with a westernized diet (Fig. 3c).

We then compared the gut microbiomes of asymptomatic RMs, chronic diarrhea RMs, humans with IBD (i.e., CD and UC, with a westernized diet), acute diarrheal humans (from rural and urban populations), and healthy humans (i.e., non-westernized and westernized diet). The PCoA plot based on Bray-Curtis distances of genus-level relative abundance profiles indicated that these samples of humans were largely clustered by diet rather than disease (Fig. 3d). Furthermore, the samples of rural acute diarrhea humans and urban acute diarrhea humans were distant in the PCoA plot. The samples of chronic diarrhea RMs were closer to the samples of rural diarrhea humans and humans with a non-westernized diet (Fig. 3d and Fig. S3c).

### The ARGs and resistant phenotype of RM gut microbiome

**Fig. 4:**
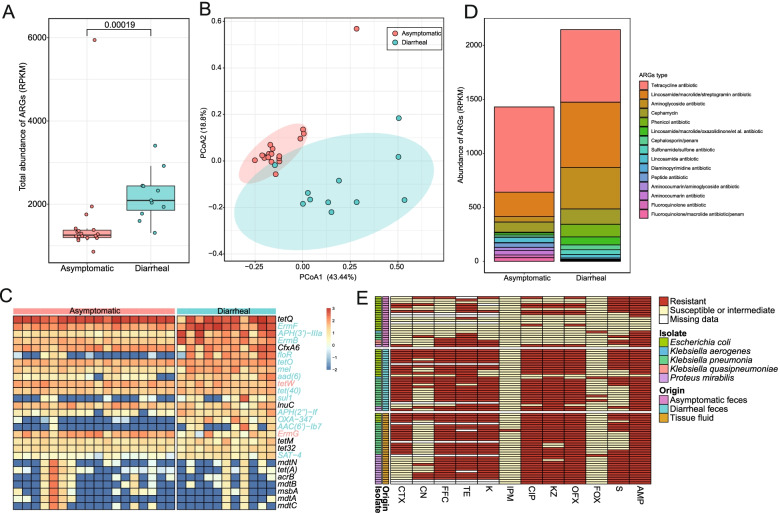
The antibiotic resistome of chronic diarrhea RMs and asymptomatic RMs. **a** Total abundance comparison of ARGs per metagenome based on the ARGs’ marker (Wilcoxon**’s** rank**-**sum test *p*-value < 0.001). **b** PCoA plot of Bray-Curtis distances of ARGs abundance in the gut of chronic diarrhea RMs and asymptomatic RMs (adonis, *R*^2^ = 0.26893, *p* = 0.001). **c** The heatmap of the prevalent ARGs in the gut of chronic diarrhea RMs and asymptomatic RMs. The red ARGs’ name means the significantly higher abundance in the gut of asymptomatic RMs and green ARGs’ name means the significantly higher abundance in the gut of chronic diarrhea RMs. **d** The prevalent ARGs type in the gut of chronic diarrhea RMs and asymptomatic RMs. **e** The antibiotic resistance profile of isolates from feces and tissue fluid of RMs. CTX, cefotaxime;CN, gentamycin; FFC, florfenicol; TE, tetracycline; K, kanamycin; IPM, imipenem; CIP, ciprofloxacin; KZ, cefazolin; OFX, ofloxacin; FOX, cefoxitin; S, streptomycin; AMP, ampicillin

The ARGs in the RM gut microbiome were identified and quantified using ShortBRED based on created unique markers. The total abundance of ARGs (measured by RPKM) in chronic diarrhea RM guts were significantly greater than in asymptomatic RM guts based on Wilcoxon’s rank-sum test (*p* = 0.00019) (Fig. 4a). The PCoA plot based on ARGs abundance showed that almost all of the samples of asymptomatic RMs and chronic diarrhea RMs were spatially separated (Fig. 4b). The *tet*Q gene encoding tetracycline antibiotic resistance was most prevalent in the gut microbiomes of asymptomatic RMs and chronic diarrhea RMs (Fig. 4c and Fig. S4a). The *tetO* and *tet(40)* encoding tetracycline antibiotic resistance; *ermF*, *ermB*, and *mel* encoding macrolide antibiotic resistance; *aph(3’)-IIIa*, *aph(2”)-If*, *aac(6’)-Ib7*, and *aad(6)* encoding aminoglycoside antibiotic resistance; *OXA-347* encoding β-lactam antibiotic resistance; *floR* encoding phenicol antibiotic resistance; *sul1* encoding sulfonamide antibiotic resistance; and *SAT-4* encoding nucleoside antibiotic resistance were significantly more abundant in the gut microbiome of chronic diarrhea RMs than asymptomatic RMs. These main classes of ARGs were tetracycline, lincosamide/macrolide/streptogramin antibiotics, aminoglycoside antibiotics, cephamycin, and phenicol antibiotics in the gut microbiome of RMs (Fig. 4d).

We analyzed the correlation between the abundance of bacteria and ARGs (correlation coefficient > 0.7, Fig. S4b). Proteobacteria and Firmicutes were the main phyla associated with ARGs. *Escherichia*, *Acinetobacter*, *Shigella*, and *Enterobacter* from Proteobacteria and *Kurthia* from Firmicutes were associated with about 50 ARGs, mainly beta-lactamases ARGs, fluoroquinolone ARGs, and efflux pump genes. Additionally, *Melaminivora*, *Sphingopyxis*, *Stenotrophomonas*, *Lysobacter*, and *Xanthomonas* from Proteobacteria and *Sphingobacterium* from Bacteroidetes were associated with about 20 ARGs, particularly aminoglycoside ARGs.

To further verify antibiotic resistance, we tested resistant phenotypes of isolates from feces and tissue fluid of captive RMs. A total of 31 *Escherichia coli* isolates, 29 *Klebsiella pneumonia* isolates, 16 *Proteus mirabilis* isolates, 2 *Klebsiella aerogenes* isolates, and 2 *Klebsiella quasipneumoniae* isolates were isolated from feces of chronic diarrhea RMs, feces of asymptomatic RMs, and tissue fluid of a dead diarrheal RM (Table S2). Almost all of the isolates from feces and tissue fluid of chronic diarrhea RMs were resistant to FFC (49/51), TE (48/49), K (48/51), CIP (55/58), KZ (54/58), OFX (55/58), and AMP (57/58) (Fig. 4e). However, the majority of these isolates were susceptible to IPM (57/58) and FOX (54/58). The isolates from feces of asymptomatic RMs had the highest resistance rate to AMP (16/22), followed by TE (12/18) and S (10/22). Similarly, almost all of these isolates were susceptible to IPM (22/22) and FOX (21/22).

## Discussion

In this study, we observed a distinct difference between the gut microbiome of chronic diarrhea RMs compared to asymptomatic individuals. Age and gender did not significantly influence the gut microbiome of these captive RMs, which had the same diet and similar living environment. Therefore, the gut microbial composition of these RMs was significantly influenced by diarrheal disease rather than age or gender. Here, we revealed the significant differences in the composition, metabolic pathways, and resistome of the gut microbiome between chronic diarrhea RMs and asymptomatic individuals.

We found that *Lactobacillus* constituted 42.4% of gut bacteria of asymptomatic RMs, which was significantly greater than 5.2% in chronic diarrhea RM guts. *Lactobacillus* spp. are important beneficial bacteria in the guts of humans [66] and healthy mammals, such as pigs [67], dogs [68], and cows [69]. Previous studies have demonstrated that *Lactobacillus* spp. were capable of inhibiting the excessive growth of opportunistic pathogens in guts through competing with opportunistic pathogens to prevent them from adherence and producing various antibiotic factors, such as bacteriocin, organic acid, and hydrogen peroxide [70–73]. Therefore, *Lactobacillus* has been considered an important probiotic for prevention and therapy of colonic infections in clinical practice [73]. In our study, *L. johnsonii*, *L. reuterii*, and *L. amylovorus* were the main *Lactobacillus* species in asymptomatic RM guts and had genes encoding mucus-binding proteins, which conferred an adhesion ability to the intestinal epithelial cell [74, 75]. The effective adherence in the intestinal tract is regarded as the basis of probiotic effects involved immunoregulation of hosts and inhibition of opportunistic pathogens [76, 77]. *Lactobacillus reuterii* and *L. amylovorus* had genes encoding bacteriocin. The bacteriocin of *Lactobacillus* can inhibit the growth of other bacteria in the guts [78]. The gene annotation of *L. johnsonii*, *L. reuteri* and *L. amylovorus* identified key lactate dehydrogenases, which catalyze pyruvate to lactate in anaerobic glycolysis [19, 79]. These characters of *Lactobacillus* implied there were effective probiotic functions in RM guts to maintain the stability of the gut microbiome and alleviate the inflammation of the gut [80, 81]. These prevalent *Lactobacillus* spp. in RMs can be considered potential probiotics to prevent diarrhea of RMs [82]. Therefore, the scarcity of *Lactobacillus* in diarrheal RM guts might be one of latent causes of diarrhea and leads to weakening of the inhibition function to other opportunistic pathogens, further causing disorder of the gut microbiome. Alternatively, the chronic diarrhea disease in RMs might cause a decrease of *Lactobacillus* in the gut.

In addition, we found that *Helicobacter* spp. primarily existed in the guts of asymptomatic RMs in accordance with a previous study [22]. For instance, *H. cinaedi* was reported to widely colonize captive RM guts [83]. Reportedly, *Helicobacter* spp. are also associated with the diarrhea of RMs [3, 84]. Therefore, *Helicobacter* spp. in RM guts might be pathogenic when the hosts were in a specific status, such as immunocompromised or infected by over-abundant *Helicobacter*. We also found that mucin-degrading bacteria were greater in chronic diarrhea RM guts. A key mechanism of diarrhea is a decrease in mucin layer thickness that exposes the intestinal epithelial cells to pathogens [85]. These mucin-degrading bacteria might speed the decrease of mucin layer thickness to cause diarrhea of RM [86].

Our finding of more abundant CAZymes in asymptomatic RM guts may indicate more active gut microbiome metabolism of carbohydrate degradation, in particular GHs and PLs, which are the key enzyme families degrading carbohydrates through hydrolysis and non-hydrolytic cleavage, respectively [87]. The functional comparison of the gut microbiome also identified a metabolic pathway enriched in the production of SCFAs in the asymptomatic RM gut microbiome. The acetate, propionate, and butyrate are the main members of SCFAs, which also include lactate, formic acid, and succinic acid [19]. Indigestible polysaccharides can be fermented by anaerobic microbiota to SCFAs in the colon [21]. The SCFAs play many key roles in regulating anti-microbial immunity and relieving gut inflammation of animals through inhibiting the growth of pathogens and activating SCFA receptors to increase regulatory T cells [20, 88–90]. The inhibition or decrease of SCFAs in animal guts may cause diarrhea while the increase of SCFAs may protect gut mucosa to decrease diarrhea [91, 92]. Meanwhile, the enriched metabolic pathway of aerobactin biosynthesis, largely due to *E. coli*, might be an important characteristic of diarrhea disease. Aerobactin, as a virulence factor, may be an important factor in extracellular infection because it can independently provide iron for pathogens to invade tissues [93, 94]. *Escherichia coli* is the main contributor of aerobactin in chronic diarrhea RMs. Consequently, *E. coli* might play an important pathogenic role in the diarrhea of RMs.

Due to the close phylogenetic relationships between humans and NHPs, there are many similarities in their genomes, physiology, and immune system [95]. The captivity of NHPs might shape a similar gut microbiome to modern humans [96], but there were still differences in the gut microbiome between captive NHPs and modern humans with different diets. We found a greater similarity in gut microbial composition between NHPs and humans with a non-westernized diet. A westernized diet consisted of a high-fat and low-fiber diet, while humans with a non-westernized diet consumed higher levels of fiber and lower levels of fat [96, 97]. Nevertheless, the opposite dietary features shaped the different gut microbiome [97]. Since captive NHPs have a similar diet to humans with a non-westernized diet, this might highly influence the similarities in gut microbiome between captive NHPs and humans with a non-westernized diet [96, 98, 99]. However, the human gut microbiome was not consistently similar across all NHP species, with the captive *P. troglodytes* gut microbiome being most similar to the gut microbiome of humans. This higher similarity in gut microbiome composition may be due to the closer phylogeny and similar diet [100, 101].

Although the symptoms of chronic diarrhea of RMs are similar to UC disease in humans [30], the gut microbiome compositions of them were obviously different in our study. The differences may be caused by these humans with UC disease consuming a westernized diet, because the gut microbiome of RMs was more similar to humans with a non-westernized diet than humans with a westernized diet. We did not collect samples of the humans with IBD consuming a non-westernized diet, but we found that the gut microbiome of chronic diarrhea RMs was more similar to rural diarrheal humans and humans with a non-westernized diet. Therefore, due to more similarities in gut microbiomes, chronic diarrhea RMs could be more suitable models in diarrheal research of rural dwellers or humans with a non-westernized diet.

The frequent use of antibiotics has resulted in antibiotic selection pressure causing the prevalence of antibiotic resistance bacteria [102]. We found that there were most abundant tetracycline ARG in RM guts and most isolates from RM guts were resistant to tetracycline. Although tetracycline antibiotics were not used in the treatment of diarrhea of RMs, long-term and widespread use of tetracycline antibiotics has caused an extensive global tetracycline resistance [103]. *Bacteroides* spp. are reportedly the main carriers of the *tetQ* gene [104, 105]. Therefore, a prevalence of *tetQ* in RM guts might be due to abundant *Bacteroides*. Similarly, the widespread aminoglycoside ARGs in the chronic diarrhea RM gut microbiome may have resulted from the use of aminoglycoside antibiotics, and it also led to a high resistance rate to kanamycin. The use of florfenicol antibiotics may have caused a high abundance of *floR* in the guts of RMs [106]. Therefore, abundant ARGs in the guts of chronic diarrhea RMs may be strongly associated with the frequent use of antibiotics.

The testing of resistant phenotypes of isolates further demonstrated serious antibiotic resistance of chronic diarrhea RMs gut microbiome. Not only was there a high abundance of ARG in the gut microbiome of chronic diarrhea RMs, but these RM guts also had high antibiotic resistance rates within isolates. These isolates from chronic diarrhea RMs generally had higher antibiotic resistance rates to florfenicol, tetracycline, aminoglycoside, quinolone, and cephalosporin antibiotics according to our susceptibility testing. Most of these antibiotics were used in previous treatments of diarrhea. Therefore, a directed use of antibiotics in the treatment of diarrheal RMs is necessary to avoid more serious antibiotic resistance. It is worth noting that almost all isolates were susceptible to imipenem and cefoxitin. Due to a stronger stabilization compared to other cephalosporins, bacteria usually had low resistance rates to cefoxitin (a type of cephamycin antibiotics) and imipenem (a type of carbapenem antibiotics) [107, 108]. Therefore, cephamycin and carbapenem antibiotics may be effective to treat diarrhea in RMs and provide new treatment protocols. Nonetheless, the use of cephamycin and carbapenem antibiotics still needs to be treated with caution to avoid novel antibiotic resistance. It is also necessary to monitor resistance phenotype(s) of common antibiotic resistance bacteria, such as *Escherichia coli*, in RM guts and the captive environment. These measures could effectively decrease the emergence and spread of antibiotic resistance in captive RMs.

## Conclusions

In conclusion, our results indicated that individuals with chronic diarrhea had significantly different gut microbiomes compared with asymptomatic individuals. We also identified that frequent use of antibiotics caused antibiotic resistance in RM guts, with serious consequences for the treatment of chronic diarrhea. According to our results, beneficial strains of *Lactobacillus* from RM guts could be regarded as a potential probiotic to prevent or relieve RM diarrhea in future research and treatment. It was necessary to monitor antibiotic resistance of bacteria in RMs’ environments to reduce the propagative risks from ARGs and multidrug-resistant bacteria. Our results also revealed a greater similarity in gut microbial compositions between captive NHPs including RMs and humans with a non-westernized diet. This result suggests that diarrheal RM could be considered a suitable model to research diarrhea in humans with a non-westernized diet. Overall, our results provided a clearer understanding of the gut microbiome and antibiotic resistome of chronic diarrhea RMs.

## Supplementary Information

**Additional file 1: Table S1.** The summary of information of RMs. **Table S2.** The antibiotic resistant profiles of isolates from RMs

**Additional file 2: Figure S1.** The gut microbiome of chronic diarrhea RMs and asymptomatic RMs. **Figure S2.** The functional comparison of gut microbiome. **Figure S3.** The gut bacterial composition of NHPs and human. **Figure S4.** The antibiotic resistance in gut microbiome of asymptomatic and chronic diarrhea RMs.

## Data Availability

The data that support the findings of this study have been deposited into the CNGB Sequence Archive (CNSA) of China National GeneBank DataBase (CNGBdb) with accession number CNP0001810.
