# Supplementary material for: The gut microbiome and antibiotic resistome of chronic diarrhea rhesus macaques (Macaca mulatta) and its similarity to the human gut microbiome
Source: Microbiome. 2022 Feb 9;10:29. doi: 10.1186/s40168-021-01218-3 (PMC8827259; doi:10.1186/s40168-021-01218-3)
Supplement: Supplementary file 2 — Additional file 1: Table S1. The summary of information of RMs. Table S2. The antibiotic resistant profiles of isolates from RMs [file 40168_2021_1218_MOESM2_ESM.pdf]

Supplementary Materials

Table S1 The summary of information of RMs

**Sample ID**	**Gender**	**Age when sampling**	**Diarrheal**	**Used antibiotics**	**Sampling date**
Asymptomatic-1	Female	3 (sub-adult)	No	-	2019.08.01
Asymptomatic-2	Female	3 (sub-adult)	No	-	2019.08.01
Asymptomatic-3	Male	15 (geratic)	No	-	2019.08.01
Asymptomatic-4	Female	3 (sub-adult)	No	-	2019.08.01
Asymptomatic-5	Male	15 (geratic)	No	-	2019.08.01
Asymptomatic-6	Male	15 (geratic)	No	-	2019.08.01
Asymptomatic-7	Male	3 (sub-adult)	No	-	2019.08.01
Asymptomatic-8	Male	3 (sub-adult)	No	-	2019.08.01
Asymptomatic-9	Male	3 (sub-adult)	No	-	2019.08.01
Asymptomatic-10	Female	15 (geratic)	No	-	2019.08.01
Asymptomatic-11	Female	15 (geratic)	No	-	2019.08.01
Asymptomatic-12	Female	15 (geratic)	No	-	2019.08.01
Asymptomatic-13	Male	8 (adult)	No	-	2019.08.01
Asymptomatic-14	Male	7 (adult)	No	-	2019.08.01
Asymptomatic-15	Female	8 (adult)	No	-	2019.08.01

Asymptomatic-16	Female	7 (adult)	No	-	2019.08.01
Asymptomatic-17	Female	7 (adult)	No	-	2019.08.01
Asymptomatic-18	Male	8 (adult)	No	-	2019.08.01
Diarrheal-1	Male	19 (geratic)	Yes	Levofloxacin and metronidazole	2019.09.10
Diarrheal-2	Female	9 (adult)	Yes	Levofloxacin and metronidazole	2019.09.10
Diarrheal-3	Male	8 (adult)	Yes	Levofloxacin and metronidazole	2019.09.10
Diarrheal-4	Female	8 (adult)	Yes	Levofloxacin, gentamicin and cephalosporins	2019.09.10
Diarrheal-5	Male	8 (adult)	Yes	Levofloxacin, gentamicin and cephalosporins	2019.09.10
Diarrheal-6	Male	8 (adult)	Yes	Levofloxacin and metronidazole	2019.09.10
Diarrheal-7	Female	8 (adult)	Yes	Levofloxacin and metronidazole	2019.09.10
Diarrheal-8	Female	5 (sub-adult)	Yes	Levofloxacin and metronidazole	2019.09.10
Diarrheal-9	Female	2 (sub-adult)	Yes	Levofloxacin, metronidazole, florfenicol and cephalosporins	2019.09.23
Diarrheal-10	Male	3 (sub-adult)	Yes	Levofloxacin, metronidazole, florfenicol and cephalosporins	2019.09.23
Diarrheal-11	Female	3 (sub-adult)	Yes	Levofloxacin, metronidazole, florfenicol and cephalosporins	2019.09.23

Table S2 The antibiotic resistant profiles of isolates from RMs

Origin	Isolations	CTX	CN	FFC	TE	K	IPM	CIP	KZ	OFX	FOX	S	AMP
Asymptomatic feces	*Escherichia coli*	S/I	R	R	×	R	S/I	R	S/I	R	S/I	R	R
Asymptomatic feces	*Escherichia coli*	S/I	S/I	S/I	R	S/I	S/I	S/I	S/I	S/I	S/I	R	R
Asymptomatic feces	*Escherichia coli*	S/I	S/I	S/I	S/I	S/I	S/I	S/I	S/I	S/I	S/I	R	R
Asymptomatic feces	*Escherichia coli*	R	R	S/I	R	×	S/I	R	R	R	S/I	R	R
Asymptomatic feces	*Escherichia coli*	R	S/I	S/I	R	S/I	S/I	R	R	R	S/I	R	R
Asymptomatic feces	*Escherichia coli*	S/I	S/I	S/I	R	S/I	S/I	S/I	S/I	S/I	S/I	R	R
Asymptomatic feces	*Escherichia coli*	R	R	S/I	R	R	S/I	R	R	R	S/I	R	R
Asymptomatic feces	*Escherichia coli*	×	×	×	×	×	S/I	R	R	R	S/I	S/I	R
Asymptomatic feces	*Escherichia coli*	×	×	×	×	×	S/I	S/I	S/I	S/I	S/I	S/I	R
Asymptomatic feces	*Escherichia coli*	S/I	S/I	S/I	R	S/I	S/I	S/I	S/I	S/I	S/I	S/I	S/I
Asymptomatic feces	*Escherichia coli*	S/I	S/I	S/I	S/I	S/I	S/I	S/I	S/I	S/I	S/I	S/I	S/I
Asymptomatic feces	*Escherichia coli*	×	×	×	×	×	S/I	S/I	S/I	S/I	S/I	S/I	S/I
Asymptomatic feces	*Escherichia coli*	S/I	S/I	S/I	S/I	S/I	S/I	R	S/I	R	S/I	S/I	S/I
Asymptomatic feces	*Escherichia coli*	S/I	S/I	S/I	S/I	S/I	S/I	S/I	S/I	S/I	S/I	S/I	S/I
Asymptomatic feces	*Escherichia coli*	S/I	S/I	S/I	S/I	S/I	S/I	S/I	S/I	S/I	S/I	S/I	S/I
Asymptomatic feces	*Klebsiella aerogenes*	S/I	S/I	S/I	S/I	S/I	S/I	S/I	S/I	S/I	R	S/I	R
Asymptomatic feces	*Klebsiella pneumonia*	S/I	S/I	S/I	R	S/I	S/I	S/I	S/I	S/I	S/I	S/I	R
Asymptomatic feces	*Klebsiella pneumonia*	S/I	R	S/I	R	S/I	S/I	S/I	S/I	S/I	S/I	R	R
Asymptomatic feces	*Klebsiella pneumonia*	S/I	S/I	R	R	S/I	S/I	S/I	S/I	S/I	S/I	S/I	R
Asymptomatic feces	*Klebsiella quasipneumoniae*	S/I	S/I	R	R	R	S/I	S/I	S/I	S/I	S/I	R	R
Asymptomatic feces	*Klebsiella quasipneumoniae*	S/I	S/I	R	R	R	S/I	S/I	S/I	S/I	S/I	R	R
Asymptomatic feces	*Proteus mirabilis*	R	S/I	R	R	R	S/I	R	R	R	S/I	S/I	R
Diarrheal feces	*Escherichia coli*	R	R	R	R	R	S/I	R	R	R	S/I	R	R

Diarrheal feces	*Escherichia coli*	R	R	R	R	R	S/I	R	R	R	S/I	R	R
Diarrheal feces	*Escherichia coli*	R	R	R	R	R	S/I	R	R	R	S/I	R	R
Diarrheal feces	*Escherichia coli*	R	R	R	R	R	S/I	R	R	R	S/I	R	R
Diarrheal feces	*Escherichia coli*	R	S/I	R	R	R	S/I	R	R	R	S/I	R	R
Diarrheal feces	*Escherichia coli*	R	R	R	R	R	S/I	R	R	R	S/I	R	R
Diarrheal feces	*Escherichia coli*	×	×	×	×	×	S/I	R	R	R	S/I	R	R
Diarrheal feces	*Escherichia coli*	R	R	R	R	R	S/I	R	R	R	S/I	R	R
Diarrheal feces	*Escherichia coli*	R	S/I	R	R	R	S/I	R	R	R	S/I	R	R
Diarrheal feces	*Escherichia coli*	R	S/I	R	R	S/I	S/I	R	R	R	S/I	S/I	R
Diarrheal feces	*Escherichia coli*	R	R	R	R	S/I	S/I	R	R	R	S/I	S/I	R
Diarrheal feces	*Escherichia coli*	S/I	S/I	S/I	R	S/I	S/I	S/I	S/I	S/I	S/I	S/I	S/I
Diarrheal feces	*Klebsiella aerogenes*	R	R	R	R	R	S/I	S/I	R	S/I	R	R	R
Diarrheal feces	*Klebsiella pneumonia*	R	R	R	R	R	S/I	R	R	R	S/I	R	R
Diarrheal feces	*Klebsiella pneumonia*	R	R	R	R	R	S/I	R	R	R	S/I	R	R
Diarrheal feces	*Klebsiella pneumonia*	R	S/I	R	S/I	R	S/I	R	R	R	S/I	R	R
Diarrheal feces	*Klebsiella pneumonia*	R	R	R	R	R	S/I	R	R	R	S/I	S/I	R
Diarrheal feces	*Klebsiella pneumonia*	R	S/I	R	R	R	S/I	R	R	R	S/I	S/I	R
Diarrheal feces	*Klebsiella pneumonia*	R	S/I	R	R	R	S/I	R	R	R	S/I	S/I	R
Diarrheal feces	*Klebsiella pneumonia*	R	S/I	R	R	R	S/I	R	R	R	S/I	S/I	R
Diarrheal feces	*Klebsiella pneumonia*	R	S/I	R	R	R	S/I	R	R	R	R	S/I	R
Diarrheal feces	*Klebsiella pneumonia*	R	S/I	R	R	R	S/I	R	R	R	S/I	S/I	R
Diarrheal feces	*Klebsiella pneumonia*	R	S/I	R	R	R	S/I	R	R	R	R	S/I	R
Diarrheal feces	*Klebsiella pneumonia*	×	×	×	×	×	S/I	R	R	R	S/I	S/I	R
Diarrheal feces	*Klebsiella pneumonia*	S/I	S/I	S/I	R	R	S/I	S/I	R	S/I	S/I	S/I	R
Diarrheal feces	*Proteus mirabilis*	S/I	R	R	R	R	S/I	R	R	R	S/I	R	R
Diarrheal feces	*Proteus mirabilis*	R	S/I	R	R	R	S/I	R	R	R	S/I	S/I	R

Tissue fluid	*Escherichia coli*	R	R	R	R	R	S/I	R	R	R	S/I	R	R
Tissue fluid	*Escherichia coli*	R	R	R	R	R	S/I	R	R	R	S/I	R	R
Tissue fluid	*Escherichia coli*	R	R	R	R	R	S/I	R	R	R	S/I	R	R
Tissue fluid	*Escherichia coli*	R	S/I	R	R	R	S/I	R	R	R	S/I	S/I	R
Tissue fluid	*Klebsiella pneumonia*	×	×	×	×	×	S/I	R	R	R	S/I	R	R
Tissue fluid	*Klebsiella pneumonia*	R	S/I	R	R	R	S/I	R	R	R	S/I	R	R
Tissue fluid	*Klebsiella pneumonia*	R	S/I	R	R	R	S/I	R	R	R	S/I	R	R
Tissue fluid	*Klebsiella pneumonia*	R	S/I	R	×	R	R	R	R	R	S/I	R	R
Tissue fluid	*Klebsiella pneumonia*	R	S/I	R	R	R	S/I	R	R	R	S/I	S/I	R
Tissue fluid	*Klebsiella pneumonia*	R	S/I	R	R	R	S/I	R	R	R	S/I	S/I	R
Tissue fluid	*Klebsiella pneumonia*	R	S/I	R	R	R	S/I	R	R	R	S/I	S/I	R
Tissue fluid	*Klebsiella pneumonia*	R	S/I	R	R	R	S/I	R	R	R	S/I	S/I	R
Tissue fluid	*Klebsiella pneumonia*	×	×	×	×	×	S/I	R	R	R	S/I	S/I	R
Tissue fluid	*Klebsiella pneumonia*	R	S/I	R	R	R	S/I	R	S/I	R	S/I	S/I	R
Tissue fluid	*Klebsiella pneumonia*	R	S/I	R	R	R	S/I	R	S/I	R	S/I	S/I	R
Tissue fluid	*Klebsiella pneumonia*	R	S/I	R	R	R	S/I	R	R	R	S/I	S/I	R
Tissue fluid	*Klebsiella pneumonia*	R	S/I	R	R	R	S/I	R	R	R	S/I	R	R
Tissue fluid	*Klebsiella pneumonia*	R	S/I	R	R	R	S/I	R	R	R	S/I	R	R
Tissue fluid	*Proteus mirabilis*	×	×	×	×	×	S/I	R	R	R	R	R	R
Tissue fluid	*Proteus mirabilis*	S/I	S/I	R	R	R	S/I	R	R	R	S/I	R	R
Tissue fluid	*Proteus mirabilis*	S/I	S/I	R	R	R	S/I	R	S/I	R	S/I	R	R
Tissue fluid	*Proteus mirabilis*	S/I	S/I	R	R	R	S/I	R	R	R	S/I	R	R
Tissue fluid	*Proteus mirabilis*	S/I	R	R	R	R	S/I	R	R	R	S/I	R	R
Tissue fluid	*Proteus mirabilis*	S/I	S/I	R	R	R	S/I	R	R	R	S/I	R	R
Tissue fluid	*Proteus mirabilis*	S/I	S/I	R	R	R	S/I	R	R	R	S/I	R	R
Tissue fluid	*Proteus mirabilis*	S/I	S/I	R	×	R	S/I	R	R	R	S/I	R	R

Tissue fluid	*Proteus mirabilis*	S/I	R	R	R	R	S/I	R	R	R	S/I	R	R
Tissue fluid	*Proteus mirabilis*	S/I	R	R	R	R	S/I	R	R	R	S/I	R	R
Tissue fluid	*Proteus mirabilis*	S/I	R	R	R	R	S/I	R	R	R	S/I	R	R
Tissue fluid	*Proteus mirabilis*	×	×	×	×	×	S/I	R	R	R	S/I	R	R
Tissue fluid	*Proteus mirabilis*	×	×	×	×	×	S/I	R	R	R	S/I	R	R

The S/I denotes susceptible or intermediate (non-resistant); the R denotes resistant; the × denotes missing data.
