# Supplementary material for: The gut microbiome and antibiotic resistome of chronic diarrhea rhesus macaques (Macaca mulatta) and its similarity to the human gut microbiome
Source: Microbiome. 2022 Feb 9;10:29. doi: 10.1186/s40168-021-01218-3 (PMC8827259; doi:10.1186/s40168-021-01218-3)
Supplement: Supplementary file 3 — Additional file 2: Figure S1. The gut microbiome of chronic diarrhea RMs and asymptomatic RMs. Figure S2. The functional comparison of gut microbiome. Figure S3. The gut bacterial composition of NHPs and human. Figure S4. The antibiotic resistance in gut microbiome of asymptomatic and chronic diarrhea RMs. [file 40168_2021_1218_MOESM3_ESM.pdf]

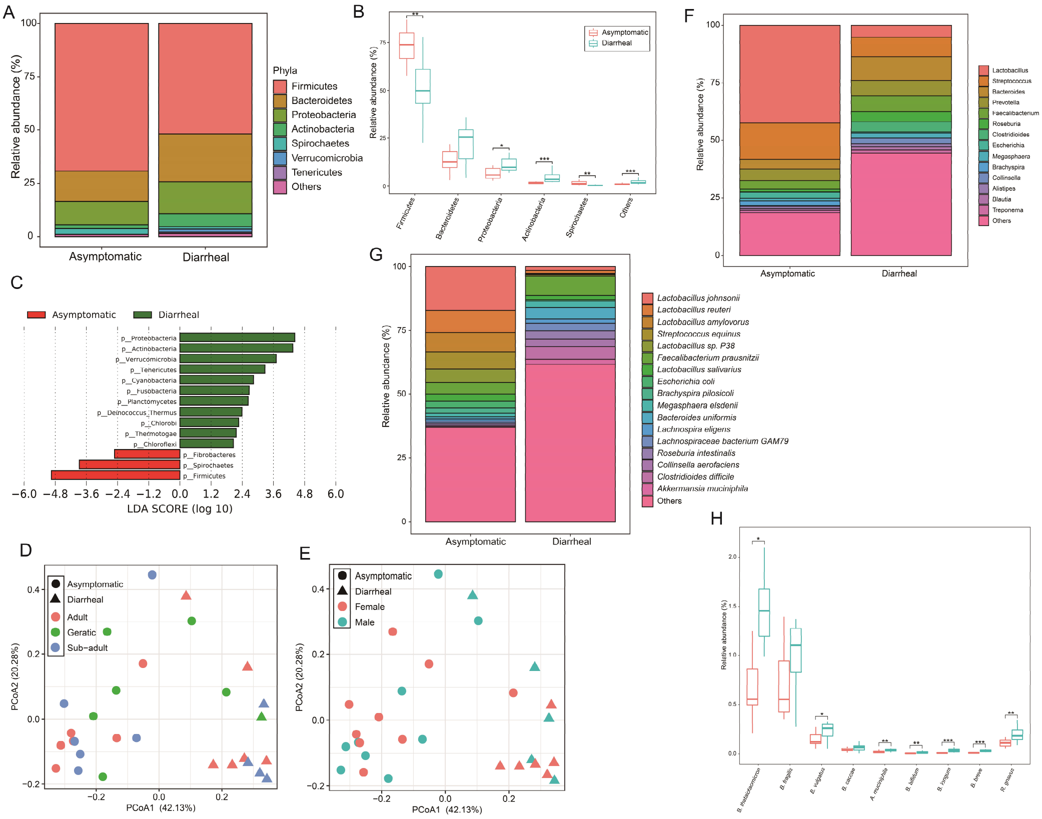

Fig. S1 The gut microbiome of chronic diarrhea RMs and asymptomatic RMs. (A) The distribution of main bacterial phyla with abundance more than 1% in all samples. (B) The comparison of main bacterial phyla. *, p-value <0.05; **, p-value <0.01; ***, p-value <0.001. (C) The difference of phylum-level relative abundance between gut bacteria of asymptomatic RMs and chronic diarrhea RMs by LEfSe ($p < 0.05$ and LDA > 3). (D) PCoA plot based on Bray-Curtis distance of genus-level relative abundance profile of RMs gut microbiomes with different ages (adonis, $R^2 = 0.04153$, $P = 0.842$). (E) PCoA plot based on Bray-Curtis distance of genus-level relative abundance profile of RMs gut microbiomes with different gender (adonis, $R^2 = 0.01849$, $P = 0.842$). (F) The distribution of main bacterial genera. (G) The distribution of main bacterial species. (H) The comparison of mucin-degradation species. *, p-value <0.05; **, p-value <0.01; ***, p-value <0.001.

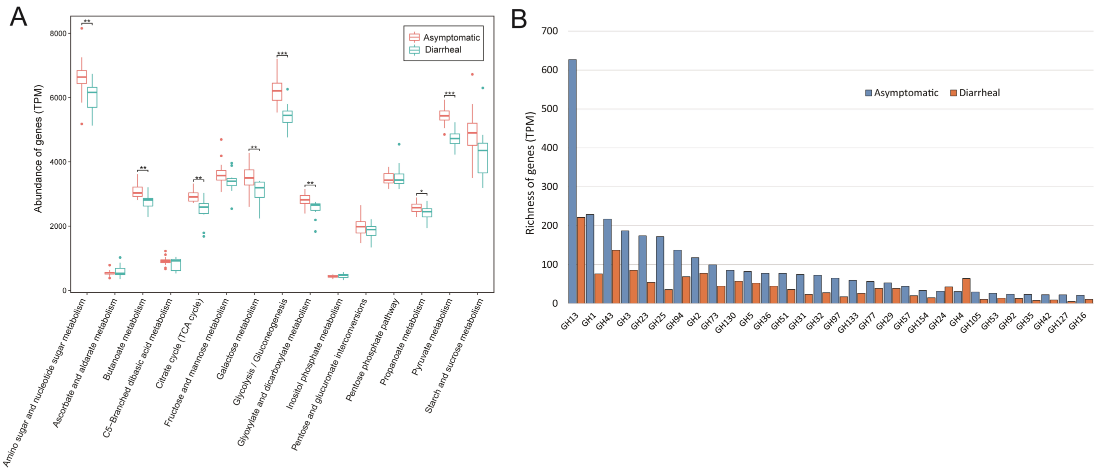

Fig. S2 The functional comparison of gut microbiome. (A) The abundance comparison of gene associated with carbohydrate metabolism. *, p-value <0.05; **, p-value <0.01; ***, p-value <0.001 (B) The abundance of main GHs family in gut.

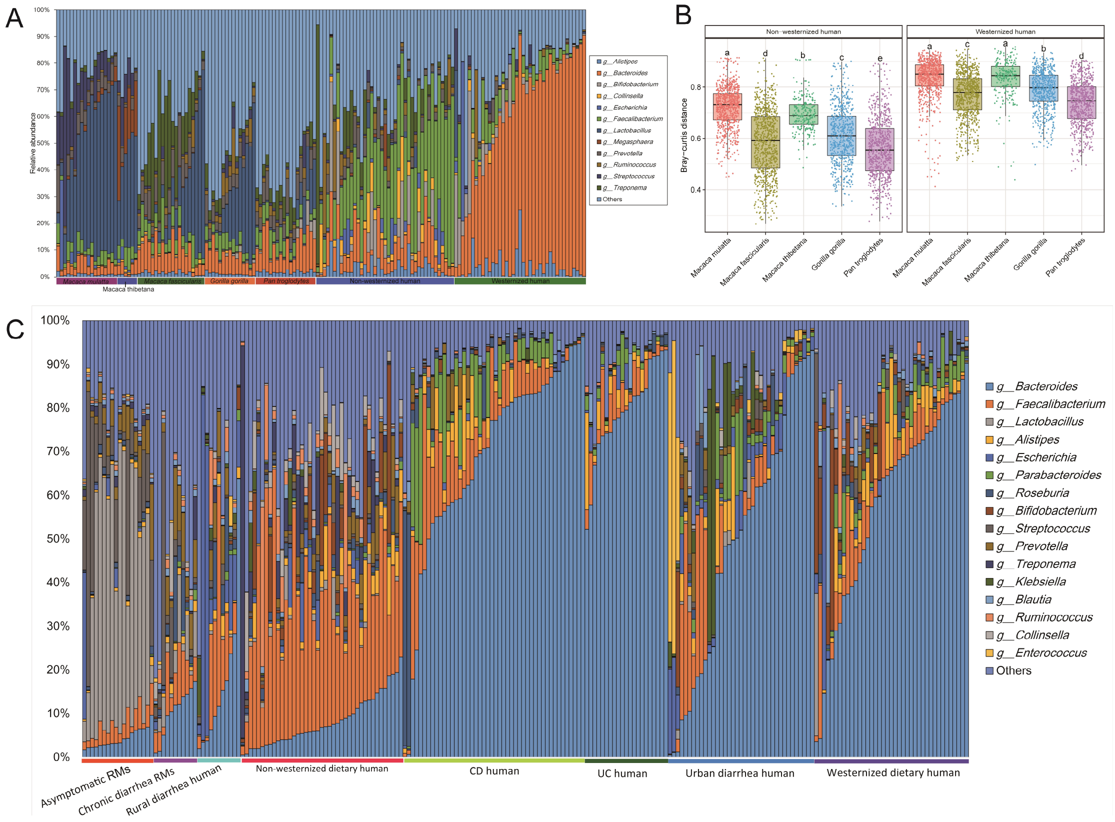

Fig. S3 The gut bacterial composition of NHPs and human. (A) The genera-level relative abundance profile of gut microbiome of NHPs and human. (B) The Bray-Curtis distance based on genus-level relative abundance of gut microbiome between NHPs and human. ***, p-value < 0.001. (C) The genera-level relative abundance profile of gut microbiome of RMs and human.

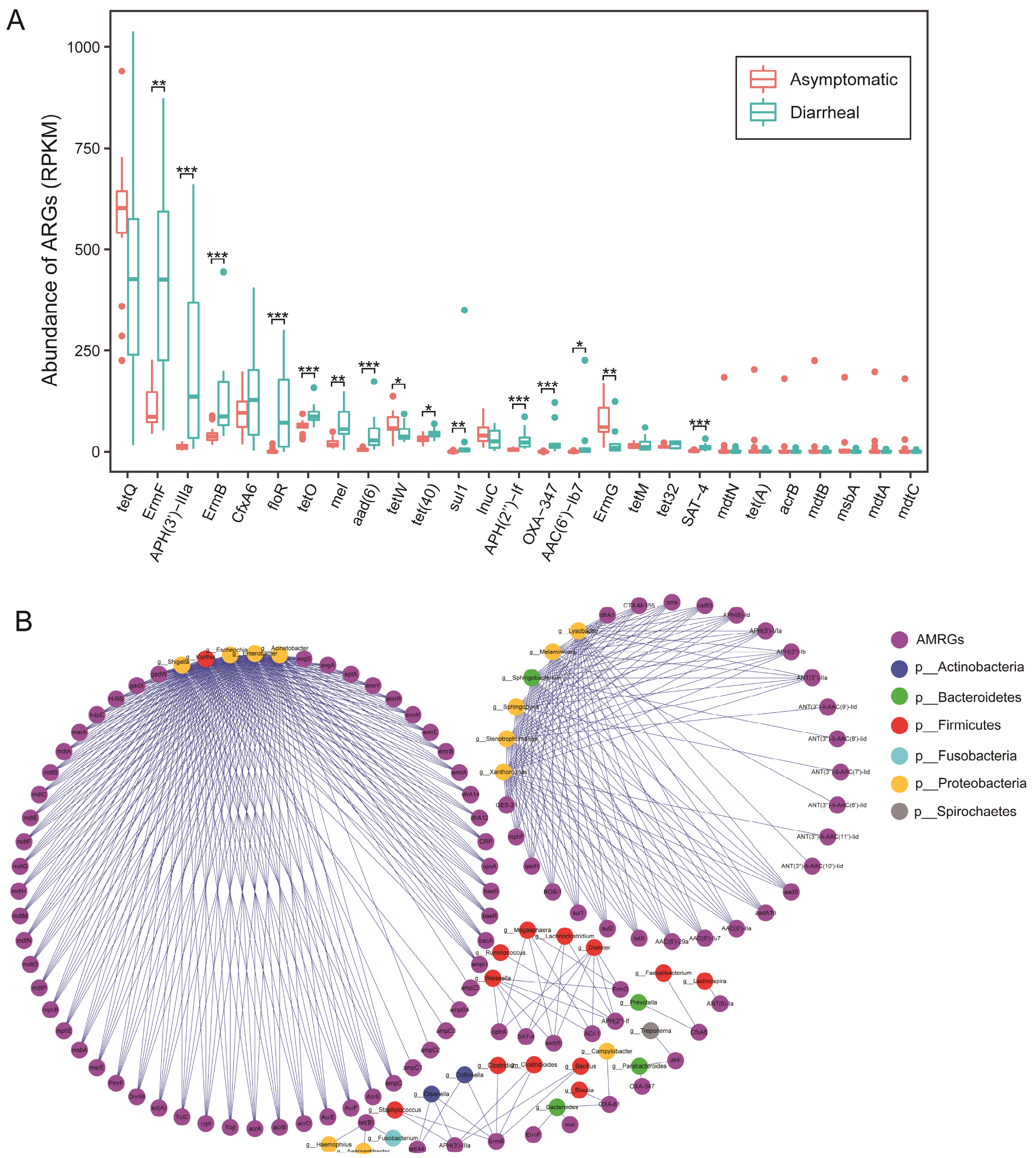

Fig. S4 The antibiotic resistance in gut microbiome of asymptomatic and chronic diarrhea RMs. (A) The abundance comparison of prevalent ARGs in gut microbiome of asymptomatic and chronic diarrhea RMs. The symbol * means $p<0.05$; the symbol ** means $p<0.01$; the symbol *** means $p<0.001$. (B) The correlation between the abundance of gut microbiome and ARGs (R2 >0.7)
